# Medical Specialists' Perspectives on the Influence of Electronic Medical Record Use on the Quality of Hospital Care: Semistructured Interview Study

**DOI:** 10.2196/27671

**Published:** 2021-10-27

**Authors:** Rube van Poelgeest, Augustinus Schrijvers, Albert Boonstra, Kit Roes

**Affiliations:** 1 Julius Center University Medical Center University of Utrecht Utrecht Netherlands; 2 Faculty of Economics and Business University of Groningen Groningen Netherlands; 3 Radboudumc University of Nijmegen Nijmegen Netherlands

**Keywords:** electronic medical record (emr), hospitals, quality, health care, medical specialist

## Abstract

**Background:**

Numerous publications show that electronic medical records (EMRs) may make an important contribution to increasing the quality of care. There are indications that particularly the medical specialist plays an important role in the use of EMRs in hospitals.

**Objective:**

The aim of this study was to examine how, and by which aspects, the relationship between EMR use and the quality of care in hospitals is influenced according to medical specialists.

**Methods:**

To answer this question, a qualitative study was conducted in the period of August-October 2018. Semistructured interviews of around 90 min were conducted with 11 medical specialists from 11 different Dutch hospitals. For analysis of the answers, we used a previously published taxonomy of factors that can influence the use of EMRs.

**Results:**

The professional experience of the participating medical specialists varied between 5 and 27 years. Using the previously published taxonomy, these medical specialists considered technical barriers the most significant for EMR use. The suboptimal change processes surrounding implementation were also perceived as a major barrier. A final major problem is related to the categories “social” (their relationships with the patients and fellow care providers), “psychological” (based on their personal issues, knowledge, and perceptions), and “time” (the time required to select, implement, and learn how to use EMR systems and subsequently enter data into the system). However, the medical specialists also identified potential technical facilitators, particularly in the assured availability of information to all health care professionals involved in the care of a patient. They see promise in using EMRs for medical decision support to improve the quality of care but consider these possibilities currently lacking.

**Conclusions:**

The 11 medical specialists shared positive experiences with EMR use when comparing it to formerly used paper records. The fact that involved health care professionals can access patient data at any time they need is considered important. However, in practice, potential quality improvement lags as long as decision support cannot be applied because of the lack of a fully coded patient record.

## Introduction

In modern-day hospitals, IT is present in many forms. Among these are information systems, networks, databases, and websites. An electronic medical record (EMR) comprehensively includes all information to support medical diagnosis and treatment within the same institution or health system. EMR use generally alludes to the transition of information to a digital form, that is, a form that can be used by electronic devices, such as computers. Various authors agree that EMRs can make an important contribution to increasing the quality of care [1,2]. However, how are EMR use and the quality of medical care related in a hospital context?

In previous studies, the two authors (RvP and KR) of this paper attempted to establish links between the extent of EMR use and the quality of medical care [3-5]. In those previous quantitative studies, they used a specially developed tool to measure the degree of EMR use in Dutch hospitals. This 8-stage (0-7) measurement tool, the so-called EMR Adoption Model (EMRAM) from the Healthcare Information and Management Systems Society (HIMSS) measures the adoption and use of EMR functions. EMRAM incorporates algorithms to score hospitals relative to their EMR capabilities and aims to encourage hospitals to use EMRs at a higher stage [6]. The HIMSS is a US not-for-profit organization dedicated to improving health care in its quality, safety, cost-effectiveness, and access through the best use of IT and management systems. It was founded in 1961 as the Hospital Management Systems Society. It is now headquartered in Chicago, IL, USA. The society has more than 80,000 individuals, 480 provider organizations, 470 nonprofit partners, and 650 health services organizations (as of December 2019). The HIMSS definition of an environment with a complete EMR (stage 7) is “an environment that is composed of the clinical data repository, clinical decision support, controlled medical vocabulary, order entry, computerized practitioner order entry, and clinical and physician documentation applications” [7]. Ultimately, the model should lead to the use of EMR systems so that the hospital no longer uses paper charts. The findings of the quantitative analysis in the previous studies [3-5] show that Dutch hospitals in 2014 particularly struggled with electronic nursing documentation. In 2012-2014, 37.5% of Dutch hospitals were unable to upload this information to the EMRs. Once this challenge is met, the next challenge for Dutch hospitals will be to equip the EMRs with closed loop medication administration (CLMA) and an advanced clinical decision support system (CDSS). The CLMA is a fully electronic medication management process in which all relevant information is seamlessly documented. All the steps in the medication cycle (ordering, verifying, preparing, and administering) are supported electronically with decision support, where relevant. A CDSS is an application that analyzes data to help health care providers make decisions and improve patient care. A CDSS focuses on using knowledge management to obtain clinical advice based on multiple factors of patient-related data. It enables integrated workflows, provides assistance at the time of care, and offers care plan recommendations.

A 2015 study [4] tried to find a correlation between the EMRAM score and Elsevier performance indicators. This yearly Elsevier publication is a Dutch nationwide publication of quality indicators for hospitals. No statistically significant correlations were found.

In the 2017 study [5], a positive association between the use of EMRs and patient quality outcomes was found for the length of stay (LOS, the duration of a single episode of hospitalization) for patients with colorectal cancer in Dutch hospitals, as measured by the Dutch Surgical Colorectal Audit (DSCA).

In a third study (2018, not yet published, available from the first author [RvP]), we did not find a significant relationship between the EMRAM score and the number of patients with adverse events (AEs), preventable AEs, AEs caused by medication, number of re-admissions (RAs), and the LOS, as measured in the NIVEL study [8]. Our research team did not understand why a better EMRAM score does not lead to a better quality of care. We believed that two intervening aspects play a role: the implementation process itself and the role of medical specialists. We believed so because of several publications on both aspects.

The first of these is a paper written by Adler-Milstein et al [9], which emphasizes the importance of the implementation process of more mature IT systems for reaching higher quality. Recent studies suggest that unsuccessful implementation of EMR systems could be due to poorly designed EMR systems, poor use of EMRs by clinicians, or social organizational aspects, such as goal conflicts, lack of time, or lack of support from colleagues [10]. The second factor is the role of the medical specialist [11,12]. Previous studies show this is an important factor in the adoption and use [6] of EMR systems in hospitals. Medical specialists are a main frontline group of users of EMR systems. In addition, whether they support and effectively use EMR systems greatly influences other user groups in the medical institution, such as nurses, pharmacists, and administrative staff. To optimize EMR use, it is therefore essential to understand what physicians perceive to be key aspects that either support or hinder the use of EMR systems, which can positively impact medical treatment and care. To substantiate our ideas about not finding a relationship between the EMRAM score and the quality of care, we undertook this study with the following open research question:

Which positive or negative aspects influence the relationship between EMR use and the quality of medical care according to medical specialists?

In this paper, the term “EMR” can concern the data themselves, the accompanying procedures, or a fundamental change in method (so-called digital transformation).

## Methods

### Design and Methodology

To answer the research question, a qualitative research study was performed. A qualitative design was considered appropriate for this question, as the primary objective was to explore more in-depth perceptions of factors and processes related to a more complex system, including social and technical components [13].

The development of EMR use in 73 Dutch hospitals in the period of 2012-2015 was measured using the EMRAM score [14]. The hospitals that were measured twice in the research period and did not work with nursing documentation in EMRs or with the CLMA and advanced CDSS were asked to participate in a follow-up study (Table 1). Two hospitals achieved a higher stage on the EMRAM score, eight hospitals stayed on the same level, and one did not respond (Table 1).

**Table 1:** Summary of participating hospitals based on the EMRAM^a^ score.

Participant number	Type of hospital	Nursing documentation and CLMA^b^/advanced CDSS^c^ in EMRs^d^ in 2012-2014	Nursing documentation and CLMA/advanced CDSS in EMRs in 2018
1	UMC^e^	No	No
2	UMC	No	Yes
3	Teaching hospital	No	No
4	Teaching hospital	No	No
5	Teaching hospital	No	N/A^f^
6	Teaching hospital	No	No
7	Local hospital	No	No
8	Local hospital	No	No
9	Local hospital	No	No
10	Local hospital	No	No
11	Local hospital	No	Yes

^a^EMRAM: Electronic Medical Record Adoption Model.

^b^CLMA: closed loop medication administration.

^c^CDSS: clinical decision support system.

^d^EMR: electronic medical record.

^e^UMC: University Medical Centre.

^f^N/A: not available.

The hospitals were approached through the chairs of their medical staff and were asked to nominate a medical specialist each to participate in this study. To be eligible, the medical specialists were required to have worked for more than 5 years in the hospital in question. Overall, the selection of participants was geared toward a balanced mix of different specialties (surgical, nonsurgical, small specialty).

In the period of August-October 2018, a semistructured interview of about 90 min was conducted with each medical specialist selected. The abovementioned research question was at the core of the interview. An item list (available from the first author [RvP]) was used by the interviewer to help the participants focus on relevant experiences in case the conversation halted. We only asked questions about aspects the medical specialists were personally dealing with; we were not interested in second-hand accounts.

For analysis of the answers, we used the classification of aspects that can influence the implementation of EMR systems based on the taxonomy of Boonstra et al [15]. This systematic literature review was carried out to identify all the barriers that result in physicians showing resistance toward EMR systems. Table 2 shows the highlights of this taxonomy model.

We used this taxonomy for the same aspects in a neutral connotation, as the same taxonomy can be followed when categorizing aspects (quotes) as facilitators of EMR use. All authors participated in the allocation of quotes from the interviews to a category of the described taxonomy, with initial allocation by the first author (RvP) and validation by the second and third authors (AJPS and AB). The results were recorded in a Microsoft Excel file, which is available upon request from the first author (RvP). Based on this classification and the primary interview recordings, the authors reached a consensus about the best way to allocate quotes to the categories of the taxonomy. All authors agreed with the final allocation of quotes.

**Table 2:** Summary of categories.

Quote category	Description
Technical	The technical aspects of the systems, the technical capabilities of the physicians and the suppliers
Psychological	Concerns regarding the use of EMRs^a^ that are based on the medical specialists’ personal issues, knowledge, and perceptions
Social	Relationships with the patients and fellow care providers but also with suppliers, insurers, and politicians
Time	Time required to select, implement, and learn how to use EMR systems and subsequently enter data into the system
Finance	Financial issues, including those related to monetary issues in implementing EMR systems
Legal	Privacy or security concerns regarding patients’ medical information
Organization	Organizational characteristics, such as size and type of individual practices
Change process	The influence of the organizational culture, incentives, community-level participation, and leadership

^a^EMR: electronic medical record.

### Ethics Approval and Consent to Participate

As this study did not involve research on human subjects, no medical ethical committee approval was required under Dutch law. Neither the Dutch Medical Research Involving Human Subjects Act (Wet Medisch-Wetenschappelijk Onderzoek met Mensen [WMO]) nor the university required ethics approval for the type of work conducted in this research. All participants orally and voluntarily agreed to participate in this study. They allowed us to use the data they provided, including quotes, under the condition of confidentiality. All participants agreed with the final report of their interviews. All participating hospitals, medical specialists, and used quotes in the manuscript were anonymized. No written permission was needed in this case.

## Results

### Participating Hospitals and Medical Specialists

In all, 11 hospitals (Table 3) agreed to participate in this qualitative study, while 3 hospitals (1 regional and 2 teaching hospitals) were unwilling or unable to participate. Each participating hospital nominated 1 medical specialist to be interviewed, representing 10 different specialties and between 5 and 27 years of experience in their current hospital. Six of them were (former) chairs of medical staff.

**Table 3:** Summary of participating hospitals and medical specialists.

Type of hospital	Number of hospitals (n)	Number of specialists (n)	Type of specialist (n)	Age (years)	Gender (n)	Number of years of experience in hospital
UMC^a^	2	732-1050	Internist (1) Anesthetist (2)	43-57	Female (1) Male (1)	10-13
Teaching hospital	4	240-377	Rheumatologist (3) Radiologist (4) Internist (5) Surgeon (6)	49-62	Male (2) Female (2)	11-23
Local hospital	5	70-187	Pediatric neurologist (7) Vascular surgeon (8) Gynecologist (9) Cardiologist (10) Pharmacist (11)	42-59	Male (5)	5-27

^a^UMC: University Medical Centre.

In total, the participants made 160 observations regarding aspects that influence the relationship between the extent of EMR use and the quality of care: 122 observations were characterized as *barriers* and 38 as *facilitators*. First, we will discuss the technical aspects that are mentioned most often. Next, we will discuss the other aspects of EMRs that influence the quality of care. Not every aspect of the taxonomy was used, because some aspects were not mentioned during the interviews. The legal aspect, related to information safety, was not mentioned in any of the interviews. The organizational aspect (type and size of the hospital) could not be addressed in the analysis because it was not part of the design of the study and was out of scope for individual participants. As explained in the Methods section, the change process aspect is of particular interest from a systems perspective, and it will be treated last to reflect on possible future developments.

The participating hospitals included two academic hospitals, four teaching hospitals, and five local hospitals (see also Tables 1 and 3). However, based on hospital type, no difference was found between participants’ observations. The availability of nursing documentation and of the CLMA and advanced CDSS in the hospital concerned did not lead to differences (see also Tables 1 and 3) in the experiences of the medical specialists.

### Technical Aspects

During the interviews, technical aspects were the category all medical specialists chose to talk about first. It therefore seems like medical specialists consider technical aspects the most important factor influencing EMR use in hospitals. To provide more insight, the related tables include a subdivision of technical aspects, followed by quotes from participants to illustrate what the more abstract terms of the model mean. A complete list of all quotes is available from the first author (RvP).

#### Customizability

Customizability is the ability of the technology system to adapt to specific needs of the user. Within the technical aspects category, customizability (Table 4) was mentioned most often, more often as a barrier than as a facilitator. The medical specialists compared EMR systems in the hospital with intelligent systems that can be used at home to buy a book or book a trip. It seems as though an administrative system has simply been converted to a medical system.

**Table 4:** Customizability: illustrative quotes from participants.

Quote type	Quote
Barrier	“Not intuitive. Terrible user interface. Unpleasant system, it clearly hasn’t been primarily designed for doctors and paramedics. An originally administrative system that has been reshaped into a medical system.” (Participant 2) “It is digital, but that about says it all. Leaves much to be desired.” (Participant 9) “We can see the added value, but these systems are shoddy. Not intuitive.” (Participant 8) “Preoperative polio. Supplementary lab research takes 1-2 days. If you want to change policy based on the results, the EMR system shows that this is impossible because the patient has not been hospitalized but is not present at the outpatient clinic either.” (Participant 2)
Facilitator	“Innumerable positive points; accessible everywhere, even at home. No more illegible notes.” (Participant 10) “Back in the day, the paper records often got lost. Lab results are available more quickly now, and the medical specialist can quickly see the daily reports of the nurses,” (Participant 7)

#### Interconnectivity/Standardization

EMR hardware and software can be used straight out of the box, but they have to be interconnected with other devices that complement the EMR system. The exchange of dossiers is often not possible due to lack of standardization, and files are often split up between different specialties because that is how the medical practice is organized in the hospital (Table 5). Getting an integral view of a patient’s situation is therefore difficult but especially important with multimorbid patients (an evergrowing group). General data, such as blood pressure, smoking, and alcohol use, are often contradictory and recorded more than once. Sometimes, multiple systems have to be simultaneously used during treatment because files are not linked—a situation that medical specialists consider potentially dangerous.

**Table 5:** Interconnectivity: illustrative quotes from participants.

Quote type	Quote
Barrier	“Many separate systems are high risk because they are not linked, for example. when transferring files.“ (Participant 11) “Gynecologists work with 4 systems (safety risk) because systems are not interlinked.” (Participant 9) “EMR^a^ now strongly split into specialties.” (Participant 4)
Facilitator	“Good-quality photos can be easily obtained.” (Participant 7) “Back in the day, there was no background information available if the GP’s^b^ notification read ‘diarrhea’; now there is.” (Participant 5)

^a^EMR: electronic medical records

^b^GP: general physician.

#### Limitations of the System

According to the participants, the IT system promises a great deal but offers little more than the old paper situation. Participants particularly point to the promised additional intelligence that is either absent from the system or present in a limited sense (Table 6). The system could offer, for example, so-called evidence-based advice based on the individual and combined patient data available in the system [16]. An oft-heard theme is also the lack of analytical tools to analyze the available data and to anticipate developments in the health of patients in the hospital.

**Table 6:** Limitations of the IT system: illustrative quotes from participants.

Quote type		Quote	
**Obsolescence: the IT system reaching its limit, becoming obsolete, and no longer remaining useful**			
	Barrier		“Actually, no added value, no decision support.” (Participant 5) “The hospital world can still learn a lot from, for example, the travel industry. It is madness that you can book a holiday in Thailand within an hour, but that scheduling an operation for a patient with a serious condition causes so many problems.” (Participant 2) About the use of analytic tools: “Executing the analysis was very time consuming. Analytics have to be carried out by an IT specialist. This makes it a hopeless affair. These tools should be included in the EMR^a^.” (Participant 9)
	Facilitator		“There is a little bit of decision support for medication (prescriptions).” (Participant 5)
**Complexity: EMRs resulting in physicians having to allocate time and effort if they are to master complexity**			
	Barrier		“Reporting of transactions [is] very complicated.” (Participant 1)
	Facilitator		N/A^b^
**Reliability: the dependability of the IT system**			
	Barrier		“In [the] case of failure of systems at polyclinic, nothing is available anymore.” (Participant 7)
	Facilitator		“Simple but works well. Very few malfunctions. Much better than paper.” (Participant 9)

^a^EMR: electronic medical record.

^b^N/A: not available.

### Other Aspects of EMRs Influencing the Quality of Care

In Table 7, other aspects of EMRs are summarized. These aspects were mentioned less often by the medical specialists but are also important influencing factors. These observations are generally consistent with the findings published elsewhere [17,18]. One thing that stands out is that medical specialists sometimes miss informal contacts of meetings with colleagues that were previously necessary due to the lack of a common digital file. Equally striking is that the financial aspect was hardly mentioned. The latter contrasts with findings in other publications [16].

**Table 7:** Other aspects of EMRs^a^: illustrative quotes from participants.

Quote type		Quote	
**Computer skills of the physician or staff: technical knowledge and skills to deal with EMRs**			
	Barrier		“Doctors are not IT savvy, [for] example, [a] radiologist who wants to look at photos at home but uses a home PC^b^ that has not been updated and therefore does not work properly.” (Participant 8)
	Facilitator		N/A^c^
**Training and support: associated with the EMR system**			
	Barrier		“The system may not be used properly by medical specialist[s]: Too little knowledge of the system Possibilities not known Defensive medicine: hedge behavior.” (Participant 4)
	Facilitator		N/A
**Psychological: personal issues, knowledge, and perceptions**			
	Barrier		“Check lists: system steers behavior. Against check lists: action is carried out anyway because I have prescribed it. Medication verification is standard procedure, so why check?” (Participant 5) “There is a contrast between old and young specialists. I think the older ones accept a limited system more easily; their demands are less high.” (Participant 7)
	Facilitator		“Enforces a certain treatment, and that is positive.” (Participant 10)
**Social: relationships with patients and fellow care providers but also with suppliers, insurers, and politicians**			
	Barrier		“Medical specialists clearly have ideas about each other. A lot of contradictions. Hard to get on the same page.” (Participant 2) “Back in the day, photos sometimes disappeared (dangerous), but medical specialists came to radiology because there was only one photo; this meant people knew each other, radiology was the center, people walked in, it used to run more smoothly. Now there is multidisciplinary consultation, but people don’t know each other anymore.” (Participant 4)
	Facilitator		“Member of medical staff (gynecologist) mans a so-called ‘wailing wall’.” (Participant 8)

^a^EMR: electronic medical record.

^b^PC: personal computer.

^c^N/A: not available.

### The Change Process Aspect

In Table 8, illustrative quotes for the change process aspect are summarized. According to the medical specialists, several preconditions for success must be met before the successful introduction of EMRs in their hospitals. There is some doubt, for example, as to whether the supplier of EMRs is willing to create links to other parts of the IT system. However, this runs counter to market forces. Moreover, the participants mentioned that governmental institutions often also still require medical specialists to use paper. A central theme for almost all interviewed medical specialists is the coded or noncoded recording of obtained information. They generally realize that encoding a medical record is a prerequisite for getting help from the EMR system based on so-called evidence-based material. Several hospitals initially started out with the recording of this information by medical specialists but later abolished this system because the medical specialists refused to work with it.

**Table 8:** The change process: illustrative quotes from participants.

The change process	Quote
Support from organizational culture	“There is too little attention for resistance in medical specialists due to, for example, time pressure.” (Participant 8) “Before, medical specialists were individual, had their own working methods. By now, a technological revolution has taken place (paper records are now electronic records). But people do not want to change (95%). They have to get out of their comfort zone. You have to invest in that. Now, medical specialists’ approach EMR^a^ as if it were paper.” (Participant 10) “The problem is that hospitals are not IT minded. Hospitals are not flexible.” (Participant 10)
Leadership	“On its own, the market will not provide properly functioning IT systems for hospitals.” (Participant 2) “Cytostatic control by pharmacies should be done via inspection on paper.” (Participant 8) “Participant sees movement from specialism based (departments) towards disease related. For example, a department of bowel cancer with [an] internist, [an] MDL^b^, [an] oncologist, and [a] radiologist. This has an impact on the way digitization is organized.” (Participant 4) “(EMR supplier mentioned) is monopolist. Does not listen to customer.” (Participant 4)
Incentives	“On its own, the market will not provide properly functioning IT systems for hospitals, for example, the market does not benefit from the exchangeability of data. Market forces therefore do not lead to the solution.” (Participant 2) “No 'reward' for 'good' use.” (Participant 8)
Participation	“Conclusion: Letting medical specialists do coding work is undesirable, but it is necessary to enable systems to ‘offer help’ on a more advanced level. This process should be structured differently by giving supporting staff a role in it.” (Participant 8)

^a^EMR: electronic medical record.

^b^MDL: *maag-darm-lever* in Dutch, meaning gastroenterologist.

## Discussion

### Preliminary Findings

To answer the research question “Which positive or negative aspects influence the relationship between EMR use and the quality of medical care according to medical specialists?”, a qualitative research study was performed.

The overall picture of the relationship between the extent of EMR use and the quality of medical care according to the participants shows that medical specialists prefer digital records over old paper ones. However, at the same time, the participants consider the technical systems old-fashioned compared to the systems they can access at home to book a trip or buy a book. The inability of all those involved (professional groups, boards, suppliers, politicians) to improve this situation is described openly by some participants. By and large, the participants do see the potential, but a better way to record coded information still needs to be found. The lack of interconnection between the different EMRs, for example, per hospital department (eg, internal medicine and cardiology), is also seen as an important limitation. Noteworthy is also that financial aspects are not mentioned often. This contrasts with other studies, in which technical issues and financial issues are mentioned in equal measure [12]. The obvious question is then whether money plays an important role according to medical specialists. Finally, these systems should be able to offer support in decision making for diagnosis and treatment [19].

The participants indicated that it is necessary to fulfil some of the preconditions for success before the EMR can make a positive contribution to the hospital’s daily practice. That is essentially the source of the medical specialist’s resistance. It takes a lot of effort and time to keep the patient file up to date. The only result is a nonpaper file, which medical specialists appreciate but is ultimately not enough to motivate them. It is tempting to make encoding medical data mandatory. However, without interventions in the organization, this is doomed to fail because many medical specialists are unwilling or unable to comply. In the end, inefficiently organized processes will then be automated at great cost and effort, while remaining inefficient at their core [20].

So, prior to the question of how to improve the available processes comes the basic question, What can be improved? Are the available business processes principally accepted, or can the search be directed toward a change in the existing processes [21]?

As stated, it is essential to understand what medical specialists perceive to be key aspects that either support or hinder the use of EMRs to positively impact diagnosis and treatment, now and in the future. These findings may help decide how medical processes can be improved with modern IT. Important in this approach is that the possibilities of modern IT, especially for advanced decision support, be taken as a starting point [22].

### Limitations of the Study

Our study had several limitations. We only interviewed medical specialists from hospitals that lacked nursing documentation in 2012-2014. These hospitals found themselves at the lowest stage of EMR use according to the EMRAM model but also had great potential to improve effectively and were able to learn from other hospitals. However, this qualitative study might still have been too early in the hospitals’ implementation of EMRs to identify aspects that are relevant for mature use of EMRs.

The age of the interviewed medical specialists varied between 42 and 62 years. These are medical specialists with a great deal of experience in the field. A question is whether the perspectives of younger medical specialists correspond with the perspectives of their older colleagues. Follow-up research might answer this question.

### Conclusions

The 11 medical specialists shared positive experiences of EMR use when comparing it to the formerly used paper records. The fact that the health professionals involved can access patient data at any time they need it is considered important. However, in practice, potential quality improvement lags as long as decision support cannot be applied due to the lack of a fully coded patient record.

## Abbreviations

AE: adverse event
CDSS: clinical decision support system
CLMA: closed loop medication administration
DSCA: Dutch Surgical Colorectal Audit
EMR: electronic medical record
EMRAM: Electronic Medical Record Adoption Model
HIMSS: Healthcare Information and Management Systems Society
GP: general physician
MDL: maag-darm-lever
NIVEL: Netherlands Institute for Health Services Research
LOS: length of stay
PC: personal computer
UMC: University Medical Centre
WMO: Wet Medisch-Wetenschappelijk Onderzoek met Mensen
